# Supplementary material for: Use of Selected Lactic Acid Bacteria for the Fermentation of Legume-Based Water Extracts
Source: Foods. 2022 Oct 25;11(21):3346. doi: 10.3390/foods11213346 (PMC9658860; doi:10.3390/foods11213346)
Supplement: Supplementary file 1 [file foods-11-03346-s001.zip › foods-1942468-supplementary.pdf]

Use of Selected Lactic Acid Bacteria for the Fermentation of Legume-Based Water Extracts

Supplementary material

Table S1. Concentration of free aminoacids (expressed in mg L^{-1}) in lupin grain based beverages at the beginning (T0) and at the end (T28) of refrigerate (4°C) storage period.

Amino Acid	*Unfermented control*		*L. acidophilus* ATCC 4356		*Lm. fermentum* DSM 20052		*Lc. paracasei* DSM 20312	
	**T0**	**T28**	**T0**	**T28**	**T0**	**T28**	**T0**	**T28**
Asp	13.01	12.22	17.76	14.95	1.97	1.66	21.43	18.84
Ser	69.58	65.44	34.59	29.23	29.15	22.47	32.82	30.16
Glu	78.37	74.67	95.63	77.07	56.93	40.36	80.83	69.12
Gly	47.77	45.56	29.88	24.38	26.61	18.75	24.96	22.50
Ala	26.42	24.69	13.55	12.41	8.00	6.61	11.56	10.62
Cys	57.11	53.63	10.80	10.36	10.96	8.99	12.67	13.10
Tyr	24.13	23.33	11.70	11.82	9.68	8.00	12.82	11.28
His	11.70	10.81	13.17	12.02	9.40	7.65	11.07	9.05
Orn	1.46	1.13	1.25	0.94	76.07	60.37	1.37	1.60
Lys	23.62	22.26	5.20	3.07	11.88	8.17	3.91	3.21
Arg	145.42	136.46	143.45	112.69	0.00	0.00	137.49	113.09
Pro	29.86	24.37	27.72	22.81	25.05	20.49	27.39	24.07
Thr	17.32	16.36	10.64	8.97	5.95	4.58	6.49	5.74
Met	9.71	9.28	1.93	1.85	0.89	0.00	0.00	0.00
Phe	20.12	17.30	4.26	3.79	3.04	2.35	5.75	5.00
Trp	6.78	5.76	6.98	5.59	6.66	6.11	7.31	6.60
Val	23.15	22.11	7.53	5.66	5.05	4.21	1.73	1.51
Ile	20.56	20.21	3.53	2.77	0.69	0.00	0.00	0.00
Leu	28.77	27.47	5.12	4.40	2.41	2.38	0.39	0.00

Table S2. Concentration of free aminoacids (expressed in mg L^{-1}) in pea grain based beverages at the beginning (T0) and at the end (T28) of refrigerate (4°C) storage period.

Amino Acid	*Unfermented control*		*L. acidophilus* ATCC 4356		*Lm. fermentum* DSM 20052		*Lc. paracasei* DSM 20312	
	**T0**	**T28**	**T0**	**T28**	**T0**	**T28**	**T0**	**T28**
Asp	11.18	10.81	16.70	14.67	7.44	6.23	20.12	17.57
Ser	55.60	58.19	41.48	39.30	34.69	31.21	48.03	43.07
Glu	118.95	113.97	118.09	115.80	118.89	89.70	137.37	110.18
Gly	34.82	33.64	47.24	38.15	66.52	52.45	47.78	40.44
Ala	127.94	132.63	142.28	131.11	146.51	126.85	143.87	127.12
Cys	44.27	44.32	0.00	0.00	0.00	0.00	0.00	0.00
Tyr	8.41	8.28	6.16	4.12	2.52	1.74	6.61	2.76
His	13.12	12.52	13.84	13.85	11.83	9.08	14.20	13.67
Orn	2.31	1.68	2.59	2.30	149.00	120.04	2.59	1.93
Lys	43.22	31.30	42.33	30.88	56.40	45.60	44.38	43.40
Arg	275.81	295.37	289.24	303.17	18.59	15.40	273.47	274.47
Pro	17.55	17.39	18.53	16.98	17.17	15.22	17.92	17.04
Thr	81.44	78.92	84.37	83.00	46.00	36.81	84.91	76.44
Met	8.53	8.44	6.38	6.14	5.04	4.075	7.13	6.29
Phe	16.10	16.49	16.10	13.90	12.27	9.73	18.23	16.28
Trp	1.75	1.67	1.80	0.00	0.94	0.00	1.95	0.00
Val	47.88	47.82	58.75	52.33	48.60	40.71	58.81	52.36
Ile	29.93	30.07	30.44	26.34	16.70	16.38	30.24	26.36
Leu	28.39	28.59	31.72	23.30	26.36	20.20	32.09	28.76

Table S3. Results of sensory analysis of lupin-based beverage before and after fermentation, allocated to macrodescriptors Appearance, Odour and Taste. In the brackets the panelist assigning the same descriptor and score. Binary response score: 0 = not expected and/or unwanted feature; 1 = expected and/or welcome feature

	**Unfermented Extract**			***L. acidophilus* ATCC 4356**			***Lm. fermentum* DSM 20052**			***Lc. paracasei* DSM 20312**		
	**Descriptor**	**Meaning**	**Score**	**Descriptor**	**Meaning**	**Score**	**Descriptor**	**Meaning**	**Score**	**Descriptor**	**Meaning**	**Score**
**Appearance**	Clear (4)	Not turbid	1	Turbid (4)	Not clear	0.25	Clear (3)	Not turbid	1	Clear (3)	Not turbid	1
	Sediment (3)	slight	0				Sediment (2)	Presence of deposit	0	Sediment (2)	Presence of deposit	0
	Yellow (3)	Pale strawyellow	1	Yellow (6)	Pale strawyellow	0.83	Yellow (5)	Nuances of pale yellow to almost transparent	0.60	Yellow (5)	Pale strawyellow	0.60
	**Average score**		**0.70**	**Average score**		**0.60**	**Average score**		**0.60**	**Average score**		**0.60**
**Odour**	Fruity (1)	melon and/or orange odours	1	Butter (1)	Butter/diacetyl	1	Fruity (2)	Fresh fruit	1	Ham (3)	Cooked ham	1
	Vegetal (4)	Grass cutting, leaves	0.75	Ham (3)	Cooked ham	1	Vegetal (2)	Grass cutting, leaves	0.50	Acid (2)	Odour pungent as vinegar	0
	Sweet/flowe (2)	Smell of fresh flowers or candy	0.5	Sandalwood (3)	Typical smell of sandalwood	1	Ham (3)	cookedham	0.67	Fruity (3)	melon and/or orange odours	1
	Almond (3)	Almond milk/horchata	1	Acid (3)	vinegar	0	Chemical (3)	Smell of pharmacy	0	Vegetal (2)	Grass cutting, leaves	0.50
	**Average score**		**0.80**	**Average score**		**0.70**			**0.50**	**Average score**		**0.70**
**Taste**	Bitter (10)	Unpleasant and persistent bitter sensation in the retrolingual areas	0	Bitter (4)	Unpleasant and persistent bitter sensation in the retrolingual areas	0	Bitter (4)	Unpleasant and persistent bitter sensation in the retrolingual areas	0	Bitter (4)	Unpleasant and persistent bitter sensation in the retrolingual areas	0
				Latex (3)	milk flavour (mixed with bitterness)	1	Liquid (2)	flowing	1	Almond (3)	almondmilk/horchata	1
				Sweet (3)	Slightly sweet	1	Vegetal (4)	Grass cutting, leaves	0.5	Vegetal (3)	Grass cutting, leaves	1
	**Average score**		**0.00**	**Average score**		**0.60**	**Average score**		**0.40**	**Average score**		**0.60**
	**Average evaluation score**		**0.50**	**Average evaluation score**		**0.63**	**Average evaluation score**		**0.50**	**Average evaluation score**		**0.63**

Table S4. Results of sensory analysis of pea-based beverage before and after fermentation, allocated to macrodescriptors Appearance, Odour and Taste. In the brackets the panelist assigning the same descriptor and score. Binary response score: 0 = not expected and/or unwanted feature; 1 = expected and/or welcome feature

	**Unfermented Extract**			***L. acidophilus* ATCC 4356**			***Lm. fermentum* DSM 20052**			***Lc. paracasei* DSM 20312**		
	**Descriptor**	**Meaning**	**Score**	**Descriptor**	**Meaning**	**Score**	**Descriptor**	**Meaning**	**Score**	**Descriptor**	**Meaning**	**Score**
**Appearance**	Sediment (1)	presence of deposit	0	Sediment (4)	presence of deposit	0.25	Sediment (3)	presence of deposit	0	Sediment (3)	Visible particles on the bottom of bottle	0
	Yellow (3)	greenish-yellow	0.70	Particulate matter (1)	Debris in the beverage	0	Turbid (3)	not clear	0.33			
	Phase separation (1)	presence of solid and liquid phase	0	Turbid (1)	not clear	1	yellow like water melon skin (4)	nuances from pale yellow to greenish	0.5	Clear (3)	Not turbid	1
	Natural (1)	remindnatural color	1	Greenish (4)	shades from opaque green to almost transparent	0.75						
	Turbid (3)	not clear	0							Yellow (4)	nuances from pale yellow to greenish	0.50
	Brilliant (1)	bright color	1									
	**Average score**		**0.40**	**Average score**		**0.50**	**Average score**		**0.30**	**Average score**		**0.50**
**Odour**	Sweet (1)	Slightly sweet	1	Vegetal (1)	grass cutting, leaves	1	Sour milk (2)	Fermented milk	0.5	Fragrant (1)	Sweet like honey or syrup	1
	Peas (5)	Fresh peas	0.60	Fruity (1)	Fresh fruit	1	Peas (4)	dry peas	0.5	Fruity (2)	Fresh fruit	1
	Vegetal (4)	Smell of hay	1	Iron (1)	Metallic	0	Vegetal (1)	grass cutting, leaves	0	Vegetal (1)	grass cutting, leaves	1
				Peas (1)	Freshpeas	1	Not fresh (1)	stale	0	Peas (1)	Fresh peas	1
				Acid (4)	Vinegar	0.75	Similiar to orange (1)	Citrus fruit	1	Floral (1)	Fresh flowers	1
				Sandal wood (1)	Typical smell of sandalwood	1	Iron (1)	metallic	0	Slightly fermented/acid (4)	weak notes of lactic fermentation	0.75
				Fermented (1)	Lactic acid fermentation	1						
	**Average score**		**0.80**	**Average score**		**0.80**	**Average score**		**0.40**	**Average score**		**0.90**
**Taste**	Light taste/watery (2)	Taste inconsistent similar to water	0,5	Light taste/watery (2)	Taste inconsistent similar to water	0.5	Acid (2)	Taste resembling diluted wine or vinegar	0.50	Light taste/watery (3)	Taste inconsistent similar to water	0,67
	Sweet (1)	Sugar	1	Natural (1)	taste resembling peas	1	Natural (1)	taste resembling peas	1	Natural (1)	taste resembling peas	1
	Vegetal (1)	Grass cutting, leaves	1	Vegetal (1)	Grass cutting, leaves	1	Fluid like water (1)	Ease to swallow	1	Sweet (1)	Sugar	1
	Natural (1)	Taste resembling peas	1	Acid (2)	Diluted vinegar	0.5	Yeast (1)	Bitter after taste	0	Fluid like water (1)	Ease to swallow	1
	Sandy (1)	Sand on the palate	0	Sandy (2)	Sand on the palate	0.5	Not sandy (1)	absence of sand on the palate	1	Acid (2)	Taste resembling diluted wine or vinegar	1
	Fluid/watery (3)	Ease to swallow	0,3	Citrus fruit (1)	Acid like diluted lemon juice	1	Citrus fruit (1)	Acid like diluted lemon juice	1	Vegetal (1)	Grass cutting, leaves	1
	Not acid (1)	Absence of acid or vinegar tastes	1	Iron (1)	Metallic	0	Vegetal (1)	grass cutting, leaves	1	Fruity (1)	Taste as fresh fruit	1
				Fluid/watery (1)	Ease to swallow	1	Iron (1)	metallic	0			
							Fermented (1)	Lactic acid fermentation	1			
	**Average score**		**0.70**	**Average score**		**0.70**	**Average score**		**0.70**	**Average score**		**0.90**
	**Average evaluation score**		**0.63**	**Average evaluation score**		**0.67**	**Average evaluation score**		**0.47**	**Average evaluation score**		**0.77**
